# Functional Corticospinal Projections from Human Supplementary Motor Area Revealed by Corticomuscular Coherence during Precise Grip Force Control

**DOI:** 10.1371/journal.pone.0060291

**Published:** 2013-03-21

**Authors:** Sophie Chen, Jonathan Entakli, Mireille Bonnard, Eric Berton, Jozina B. De Graaf

**Affiliations:** 1 Aix-Marseille Université, CNRS, ISM UMR 7287, 13288, Marseille, France; 2 Aix-Marseille Université, INSERM, INS UMR_S 1106, 13385, Marseille, France; The University of Western Ontario, Canada

## Abstract

The purpose of the present study was to investigate whether corticospinal projections from human supplementary motor area (SMA) are functional during precise force control with the precision grip (thumb-index opposition). Since beta band corticomuscular coherence (CMC) is well-accepted to reflect efferent corticospinal transmission, we analyzed the beta band CMC obtained with simultaneous recording of electroencephalographic (EEG) and electromyographic (EMG) signals. Subjects performed a bimanual precise visuomotor force tracking task by applying isometric low grip forces with their right hand precision grip on a custom device with strain gauges. Concurrently, they held the device with their left hand precision grip, producing similar grip forces but without any precision constraints, to relieve the right hand. Some subjects also participated in a unimanual control condition in which they performed the task with only the right hand precision grip while the device was held by a mechanical grip. We analyzed whole scalp topographies of beta band CMC between 64 EEG channels and 4 EMG intrinsic hand muscles, 2 for each hand. To compare the different topographies, we performed non-parametric statistical tests based on spatio-spectral clustering. For the right hand, we obtained significant beta band CMC over the contralateral M1 region as well as over the SMA region during static force contraction periods. For the left hand, however, beta band CMC was only found over the contralateral M1. By comparing unimanual and bimanual conditions for right hand muscles, no significant difference was found on beta band CMC over M1 and SMA. We conclude that the beta band CMC found over SMA for right hand muscles results from the precision constraints and not from the bimanual aspect of the task. The result of the present study strongly suggests that the corticospinal projections from human SMA become functional when high precision force control is required.

## Introduction

The highly developed ability of some primates, including humans, to perform a precision grip (thumb-index opposition) is generally accepted to require direct spinal projections from the primary motor cortex (M1), a connection termed the corticospinal (CS) pathway [1]–[4]. Direct spinal projections from secondary motor areas have also been found [5]. The medial wall of each hemisphere, where the supplementary motor area (SMA) is located, is one of the frontal areas with a large number of corticospinally projecting neurons [6], [7]. However, until now, their existence has been mainly revealed by anatomical approaches [8]–[13] and very few studies on their functional role have been reported [14]. More recently, non-invasive transcranial magnetic stimulation (TMS) studies in humans have shown motor evoked potentials in hand muscles following SMA stimulation with a latency comparable to that found for M1 stimulation [15], [16], strongly suggesting the existence of fast CS projections from SMA. However, since these TMS studies were performed with passive subjects, the functionality of the CS projections from SMA in motor control is still a matter of debate. In other words, to date it is not clear whether these CS neurons in SMA are active during force control and, consequently, contribute to muscle activity. The purpose of the present study was to investigate whether the CS projections from human SMA are functional during force control with a precision grip.

Previous studies, both in human subjects using fMRI [17]–[19] and in non-human primates using intracortical spike recording [20], reported SMA activity during precision grip and maintained force control tasks. Unfortunately, because of the exploration methods, the results reported in these studies do not identify whether SMA activity is related to activity of the neurons projecting to the spinal cord. CS neural transmission can be studied non-invasively using corticomuscular coherence (CMC), reflecting the functional coupling between cortical activity and muscle activity [21]–[24]. CMC in the beta band frequencies (14–35 Hz) has been extensively reported during maintained precise force control [25]–[30] and is now well-accepted to reflect efferent neural transmission [31]–[33]. The present study investigated the CS neural transmission from the medial frontal region, i.e. SMA, to the intrinsic hand muscles by analyzing the beta-band CMC during an isometric force contraction task.

Subjects in the present study participated in bimanual and unimanual experimental conditions. An original aspect of the bimanual protocol was the indispensable cooperation of the left hand with the right hand. In the bimanual condition, the subjects held a custom device, with both hands applying low, isometric forces. The right hand was required to apply a precise force to the device during the task, while the left hand acted as a support with no constraints on precision. Since SMA activity has often been reported to be specifically related to bimanual coordination [34]–[36], a subset of the subjects also participated in the unimanual protocol, as a control condition, in which they performed the task with only their right hand precision grip while the device was held by a mechanical grip.

In the literature, beta-band CMC has been extensively localized over the contralateral primary sensorimotor cortex but seldom over the medial frontal cortical region [37]–[39]. One of the reasons for this (others will be elaborated in the discussion section) is that most of the previous EEG studies [27], [29], [32], [40] as well as MEG studies [22], [26], [41], [42] on CMC reported results either exclusively for electrodes or sensors with the maximum CMC values, or for a pre-selected subset of electrodes or sensors, which were in both cases lying over the contralateral primary sensorimotor region. The present study analyzed the whole-scalp topography of beta-band CMC, obtained by simultaneous recording of EMG and high-resolution EEG, without any prior selection of electrodes of interest.

To investigate whether the CS projection from human SMA are functional during force control with precision grip, we analyzed the beta band CMC during a precise visuomotor force tracking task imposing isometric force production. The subjects were involved in bimanual and unimanual experimental conditions. In the bimanual condition, subjects performed different behavioural tasks with each hand with different precision constraints. We compared the beta CMC obtained for each hand using a non-parametric permutation test leading to spatio-spectral clusters for which significant differences were found. The same statistical test was used to test the difference between bimanual and unimanual conditions of the beta CMC for the right hand muscles.

## Materials and Methods

### Ethics statements

The protocol was approved by the ethics committee CPP Sud-Méditerranée II. The experiments were done with written informed consent from all the participants and conducted in accordance with the Declaration of Helsinki.

### Participants

Nine healthy right-handed volunteers (mean age, 32; range, 25–46; 5 women) with normal or corrected-to-normal vision participated in the bimanual condition. Five of them (mean age, 29; range 25–45 years, 2 women) also participated in the unimanual condition. The subjects did not have any known neurological pathology. Their right-handedness was confirmed by systematic questioning about daily manipulations.

### Experimental paradigm

**Figure 1:**
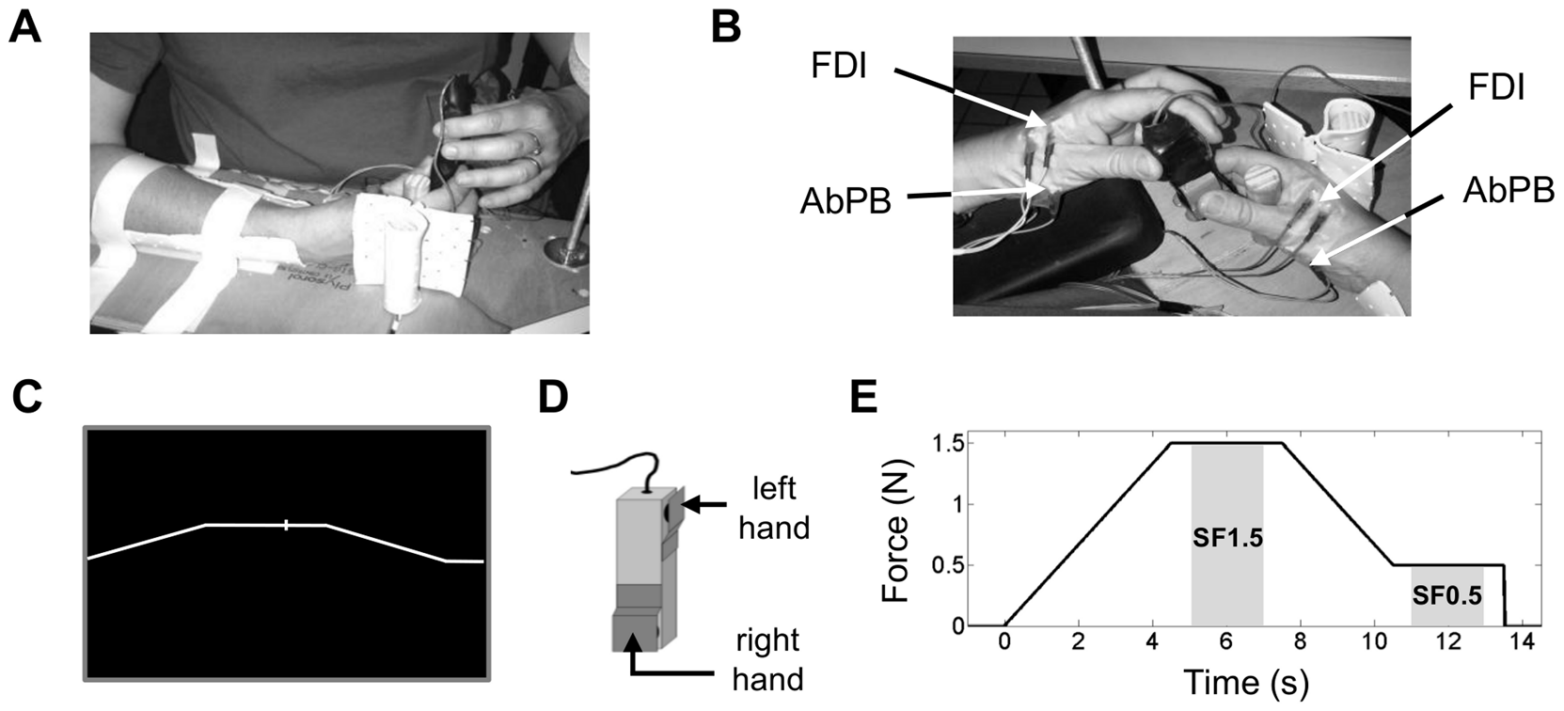
A visuomotor force tracking task imposing bimanual cooperation. The left hand held the custom device at one extremity while the right hand concurrently performed the visuomotor force tracking task by producing low forces at the other extremity of the same device. **B.** EMG was simultaneously recorded on two muscles of right and left hands, the First Dorsal Interosseus (FDI) and the Abductor Pollicis Brevis (AbPB). **C.** The target force profile for the right hand was continuously presented in a 10 s window, moving from right to left on the computer screen. The right hand force production was represented by the position of a cursor moving vertically, upwards with increasing force, 1 N corresponding to 3 cm on the screen. The horizontal position of the cursor was fixed in the middle of the computer screen. The subjects were instructed to match the vertical position of the cursor with the target force. No feedback was given concerning left hand force production. **D.** The custom device was mounted with two strain gauges to detect the grip forces produced by the right and left hands. **E.** Time course of one trial, all trials had the same force profile and duration. Each trial was was divided into 4 characteristic periods: an ascending ramp of 4.5 s with a force level linearly increasing from 0 to 1.5 N, a static force period of 3 s with a force level fixed at 1.5 N (**SF1.5**), a descending ramp of 3 s with a force level linearly decreasing from 1.5 to 0.5 N, and a static force period of 3 s with a force level fixed at 0.5 N (**SF0.5**). The present study focussed on the time periods SF1.5 and SF0.5 (gray-shaded areas).

In both the bimanual and unimanual conditions, the subjects were comfortably seated on a medical chair with both forearms resting on armrests and the neck resting on a pillow in order to avoid fatigue and excessive muscle contractions. The subjects were instructed to perform a precise visuomotor force tracking task by applying isometric low forces with their thumb-index precision grip of the right hand on a custom device with two carefully calibrated strain gauges. Both strain gauges only measured the grip force (i.e. the normal component of the force) produced by the subject. The task consisted in matching the vertical position of a cursor with a target force profile. Both the target force and the cursor were presented on a computer screen (Fig. 1C). The horizontal position of the cursor was fixed in the middle of the screen while its vertical position varied according to the grip force produced by the right hand, the cursor moving upwards with increasing force. This force was applied on one extremity of the custom device and was measured by one of the two strain gauges (as shown in Fig. 1A, D). The size of the cursor was equal to 6 mm corresponding to 0.2 N with respect to the force scale on the screen display (Fig. 1C). The target force profile moved from the right to the left side of the screen and was continuously visible in a ten-second window. Each trial comprised the same force profile with the same total duration of 13.5 s and was divided into 4 characteristic periods (Fig. 1E). Each trial started with an ascending ramp during which the target force linearly increased from 0 N to 1.5 N in 4.5 s, followed by a static force period during which the target force remained stable at 1.5 N for 3 s (henceforth named **SF1.5**). This period was followed by a descending ramp during which the target force linearly decreased from 1.5 N to 0.5 N in 3 s. Each trial ended with a static force period during which the target force curve remained stable at 0.5 N for 3 s (henceforth referred to as **SF0.5**). At the end of each trial, the target force instantly decreased from 0.5 N to 0 N and remained at 0 N for an inter-trial period of 7 s. During this latter period, the subjects could relax and move their fingers and head. The subjects performed a total of 90 or 120 trials in 3 or 4 runs of 30 trials each. Between each run, the subjects rested for several minutes to prevent fatigue. On average, the behavioural part of the experiment required a total time of 50 minutes. To avoid learning effects during the experiment, each subject was trained several days before the experiment for a minimum of 30 min.

In the bimanual condition, the subjects held the device at one extremity with their left thumb-index precision grip while the right hand performed the visuomotor force tracking task at the other extremity of the device (Fig. 1A, D). The strain gauge placed at the upper part of the device recorded the grip force of the left hand (Fig. 1D). The mass of the device was set at 84 g such that the subjects were unable to produce the very low forces of the target curve with only their right hand holding the device. If in fact the subject were to use only their right hand, the minimum grip force required to prevent the object from slipping would be higher than the maximum instructed force (i.e. 1.5 N). Hence, the crucial role of the left hand was to hold the device, thereby allowing the right hand to freely produce the low forces of the visuomotor force tracking task. As such, this task required bimanual cooperation to reach a common goal [43]. Regarding the left hand grip, subjects were asked to hold the device with a self-selected natural grip force. No feedback was given on the force production of the left hand grip. To reduce the interaction between the grip forces produced by both hands, both precision grips were oriented orthogonally (see Fig. 1A, B, D). Indeed, the orthogonal orientation of the precision grips ensures that the normal components of the forces do not interact as the dot product of two orthogonal forces is zero.

In the unimanual condition, the device was fixed by a mechanical grip which was also orthogonally oriented with respect to the right hand. The mechanical grip replaced the role of the left hand while the visuomotor force tracking task was again performed with the right hand.

Given the duration of each trial (13.5 s) and the frequency band of interest (14–35 Hz, beta band frequency), no instructions regarding eye blinking were given to the subjects. Indeed, eye-blinking artifacts have frequency components that are less than 6 Hz, whereas face muscle contraction artifacts (easily induced by restraining blinking) have frequency components that overlap with the frequency band of interest of the present study.

### Recordings

High-resolution EEG was recorded with an Advanced Neuro Technology system (ANT, Enschede, The Netherlands) using a common average reference. The electrical field was detected by 64 Ag/AgCl electrodes mounted on an elastic cap with shielded wires and positioned according to the extended 10–20 system (WaveGuard cap system of ANT). Scalp electrode impedances were maintained under 5 kΩ during the whole experiment. Surface bipolar EMG was simultaneously recorded from two muscles of each hand, the First Dorsal Interosseus (**FDI**) and the Abductor Pollicis Brevis (**AbPB**) (Fig. 1B). The ground electrode, common for EEG and EMG, was positioned on the midline of the scalp at the level of the prefrontal cortex. Both EEG and EMG signals were amplified using a full-band EEG DC amplifier powered by a battery and were recorded at a sampling rate of 1024 Hz. The grip forces of both hands were recorded simultaneously from the two strain gauges placed at the two extremities of the custom device (Fig. 1D) at a sampling rate of 100 Hz. The signal of the right hand force production was online translated into the vertical position of the cursor. All signals were saved on a hard disk for off-line analysis.

### Data analysis

All analyses were performed using MATLAB. The spectral analysis and the related statistics were calculated using FieldTrip, an open-source MATLAB toolbox for neurophysiological data analysis [44]. The duration of the different force profile periods was at least 3 s. However since the transitions (between the ramps and the stable force periods) added variability, the middle 2 s of both steady-hold periods appeared to be the most stable ones across trials. Therefore, we only considered the middle two-second window of the different periods for most of the analyses.

#### Selection of trials

The selection of correct trials was based on the performance of the right hand, and carried out for each subject individually. First, we performed a visual screening to exclude the trials during which the force production clearly differed from the target force profile. The proportion of correct samples in the middle-two-second window for each force profile period and each trial was calculated. A correct sample corresponds to a sample for which the target force was contained within the cursor. The mean and the standard deviation of the performance across trials were computed. Finally, for each force profile period, the trials with a performance lower than the mean minus twice the standard deviation were excluded. This procedure gave a selection of correct trials per period. For each subject, we selected the trials for which each force profile period was correctly performed.

#### Preliminary EMG analysis

EMG data was first analyzed in the time domain in order to determine the relative activation level of each muscle. This involved the following steps for each subject: (1) The EMG signals were off-line band-pass filtered between 10 and 450 Hz. (2) The filtered EMG signals were rectified by taking the absolute values. (3) A low-pass filter at 5 Hz was applied to the rectified data. (4) To obtain the mean EMG envelopes for each subject, the processed data were averaged across trials. Prior to averaging across subjects, the mean EMG envelop of each muscle was normalized by the maximum value of the mean EMG envelop. The filtering was performed using a bidirectional 2^nd^ order Butterworth filter.

#### EEG-EMG coherence analysis

The coherence function gives a measure of the correlation between two signals in the spectral domain and is expressed as a normalized quantity yielding values between zero and one [45], [46]. The corticomuscular coherence (CMC) is determined by calculating the coherence between each EEG and EMG channel according to the following equations.

The cross-spectrum between signals *x* and *y* at frequency *f* averaged across *N* data segments, 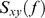 was calculated as follows:
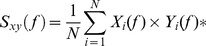
(1)where 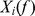
denotes the Fourier transform of the data segment *i* of the channel *x* at frequency *f*, and 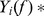 denotes the complex conjugate of the Fourier transform of the data segment *i* of the channel *y* at frequency *f*. When *x* = *y*, 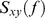 denotes the auto-spectrum of the signal.

The coherence between signals *x* and *y* at frequency *f*, 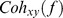 is calculated with values obtained from (1) according to the following equation:
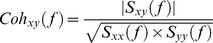



As a preprocessing step, both raw EMG and EEG signals were high-pass filtered at 1 Hz (bidirectional 2^nd^ order Butterworth filter). Subsequently, the filtered EMG signals were rectified by computing the absolute value of the Hilbert transform [47]. For the present study, we focused on the middle two-second window of the two static force periods (SF1.5 and SF0.5) (see Fig. 1E). The auto-spectra and cross-spectra were computed using the multitaper method [48]. For the time-dependent estimation of cross-spectra and auto-spectra, we used a sliding time window of 0.4 s with a sliding step of 0.05 s. Therefore, the SF0.5 and SF1.5 periods were cut into 40 overlapping segments. Each data segment was tapered using a set of discrete prolate spheroidal sequences (DPSS). Following the recommendation made by Schoffelen and collaborators [49] concerning the beta frequency band, for the cross-spectra and the auto-spectra estimations we used three tapers for each frequency bin, which leads to a spectral smoothing of ±5 Hz. The time-dependent estimation of cross-spectra and auto-spectra were then averaged accros correct trials prior to computing the time-dependent CMC. For the whole-scalp CMC analysis, power spectra and cross spectra were averaged across data segments of SF1.5 and SF0.5 periods respectively, and across the correct trials, prior to computing the CMC. These force profile-specific CMC values were then used for statistical comparisons (see Statistical analysis section).

#### Selection of sensors of interest

To restrain our statistical analysis to a subset of EEG-EMG channel pairs, we defined subsets of sensors of interest based on the topographies of the spatially Z-scored CMC. These normalized CMC values will be referred to as **z-score CMC.** This procedure was formerly used and described by Schoffelen and collaborators [49] and is necessary prior to averaging across the subjects since the CMC values are subject-specific [50].

For each subject and for a given muscle, in the time-frequency estimation of coherence of each EEG-EMG channel combination, we calculated the z-score CMC of each time-frequency bin, according to the following formula:
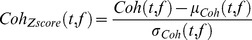
where
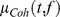
denotes the average across the CMC of the 64 EEG-EMG channel combinations for a given muscle and time-frequency bin and 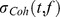 denotes the standard deviation across the CMC of the 64 EEG-EMG channel combinations for a given muscle and time-frequency bin. The topographies used to define the regions of interest were obtained after averaging the z-score CMC within each of the SF1.5 and SF0.5 periods, and over the beta frequency band defined around the maximum values, i.e., between 20 and 30 Hz for this study (see the Results section). Following this analysis, we defined subsets of EEG-EMG channel pairs as regions of interest.

#### Statistical analysis

The statistical inference on the CMC was performed at the subject group level using a non-parametric permutation test based on clustering in the spatio-spectral domain. This cluster-based statistical method required a Z-transformation of the CMC values calculated according to the following formula and refered to as **z-spectra CMC** [49], [51]. The z-spectra CMC 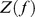 was calculated for each frequency bin:
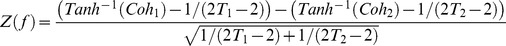
where Coh_n_ is the CMC value in condition *n*, T_n_ is the number of tapers used for the spectral estimation of the data in condition *n*. T_n_ depends on the quantity of data used to calculate the CMC, and is defined according to: 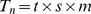 where 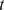 is the number of tapers used per frequency bin (equal to 3 in the present study), 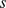 is the number of segments of data per trial and condition, 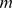 is the number of trials considered per subject. We tested whether the Z-spectra CMC of the condition difference were significantly different from 0 in the whole 14–35 Hz frequency band. The Monte Carlo approximation was performed by computing 5000 permutations for each comparison. The significance level was set to a two sided p-value of 0.05 for both first-level and second-level test statistics. Depending on the conditions to be compared, we defined different sets of electrodes: For the comparisons between conditions involving the muscles of same hand, we took the electrodes lying over the hand area of the contralateral motor cortex (M1_R_ or M1_L_) and over the supplementary motor area (SMA). For the comparisons between the left and the right hand muscles, we considered a pool of electrodes lying over both motor cortices (M1_R_ and M1_L_) and SMA. Regarding the group of subjects, for comparison within the bimanual condition, we considered the nine subjects. For comparison between unimanual and bimanual conditions, we considered the five subjects who participated in both.

## Results

### Behavioural performance

In the bimanual condition, on the average 82.3% of the trials were identified as correct trials with performance average of 96% during SF1.5 and 89.2% during SF0.5. In the unimanual condition, 84.9% of the trials were identified as correct trials with performance average of 97.1% during SF1.5, and 93.9% for SF0.5. No difference in performance of the right hand was found between the bimanual and unimanual conditions (Wilcoxon signed rank test; p>0.05).

**Figure 2:**
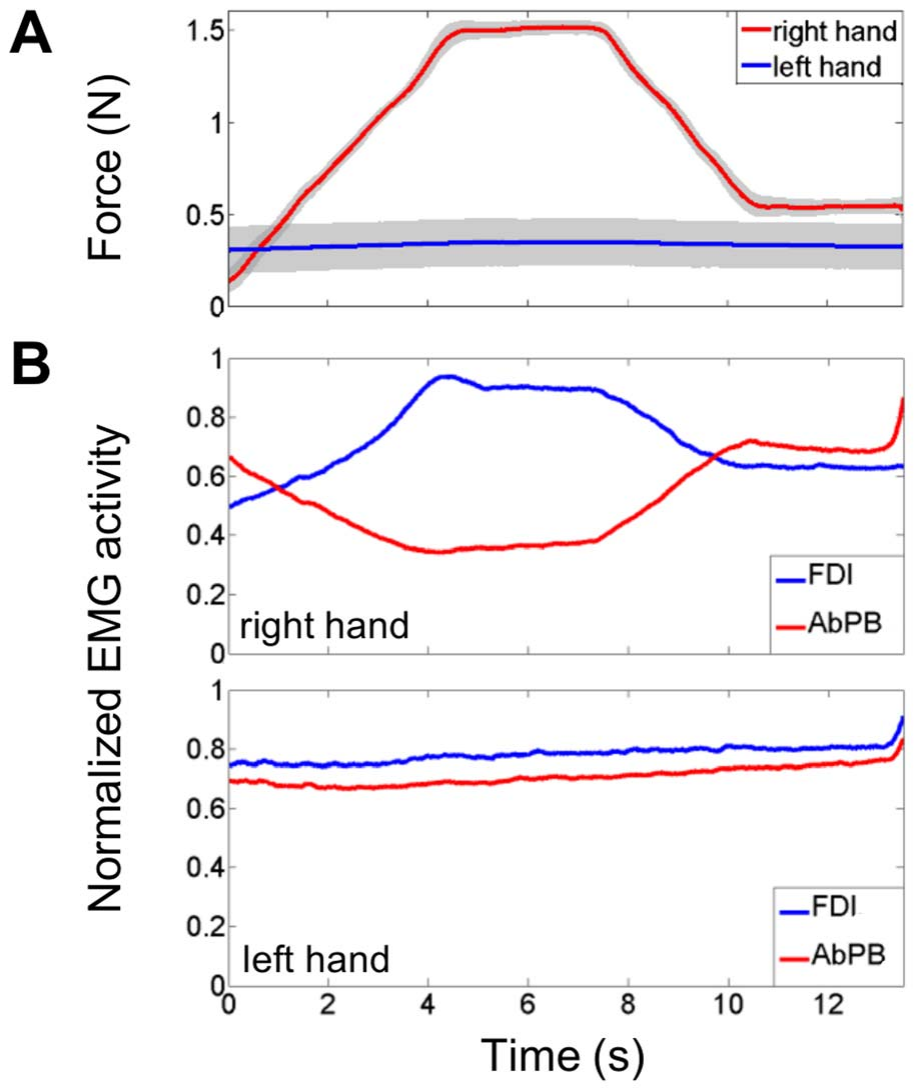
Force production and normalized muscle activation levels for both hands in the bimanual condition. **A.** Grand average time-dependent plots of the force production of the right hand (in red) and the left hand (in blue). The grand average between-trial variability at each time point is represented by the gray-shaded areas. **B.** Grand average normalized EMG activity envelop obtained for the right hand (upper panel) and left hand (lower panel) muscles: FDI (in blue) and AbPB (in red). The vertical axis represents the normalized EMG activity; the horizontal axis represents the time scale ranging from 0 to 13.5 s with 0 s, off-scale, corresponding to the beginning of the ascending ramp.

**Figure 3:**
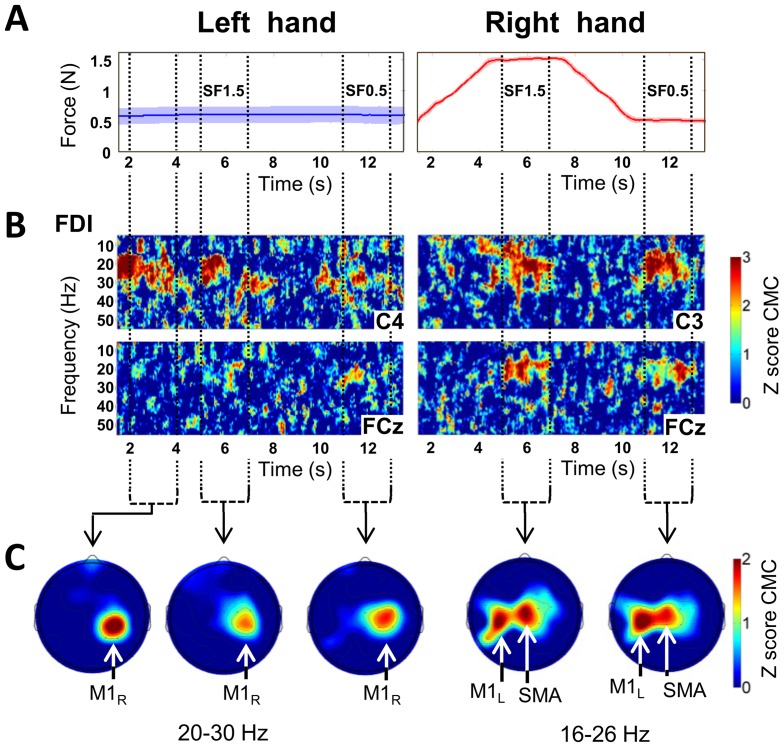
Behavioral and electrophysiological results for a typical subject in the bimanual condition. **A.** Time-dependent plots of the force production and between-trial variability at each time point for left (in blue) and right (in red) hands. The vertical dotted lines indicate the time windows over which we averaged to obtain the topographies. **B.** Time-frequency plots of the z-score CMC between left hand FDI and electrodes C4 and FCz (left side), and between right hand FDI and electrodes C3 and FCz (right side). The color scale indicates the z-score CMC value, thresholded between 0 and 3 for clarity. The vertical axis represents the frequency scale ranging from 10 to 50 Hz. The horizontal axis represents the time scale ranging from 1.5 to 13.5 s (0 s, off-scale, corresponds to the beginning of the ascending ramp). **C.** Z-score CMC topographies averaged over the frequency band 20–30 Hz for left hand, over the frequency band 16–26 Hz for right hand, and over 2 s of the periods of interest SF1.5 and SF0.5, respectively. For the left hand, an additional topography averaged over 2 to 4 s after the beginning of the trials is shown. The color scale indicates the z-score CMC values, thresholded between 0 and 2 for clarity. *M1_R_*: right primary motor cortex. *M1_L_*: left primary motor cortex. *SMA*: supplementary motor area.

Figure 2A shows the grand average time-dependent plot of the grip forces exerted in the bimanual condition by the right hand (in red) and the left hand (in blue). As was expected, the force production pattern of the right hand matched the target force profile. The force production of the left hand was essentially stable and varied on the average between 0.30 and 0.36 N. To study the between-trial variability, the standard deviation for each time sample across the correct trials was calculated for each subject and for each hand. The grand average between-trial standard deviation is represented by the gray-shaded areas in Figure 2A. It can be seen that the between-trial variability of the left hand is higher than that of the right hand. All these observations also hold for individual subjects. The results for a typical subject are shown in Figure 3. One can notice in Figure 3A that the left hand force production is stable and close to 0.5 N with a higher between-trial variability than that of the right hand. Some other subjects showed even higher between-trial variability with left hand force production ranging from 0.5 N to 1.5 N across trials (not shown).

### EMG activity patterns

The grand average of the normalized EMG envelop for each muscle (FDI in blue, AbPB in red) for the right and the left hands, obtained in the bimanual condition is shown in Figure 2B. For the right hand, the pattern of the FDI activity (in blue) followed the target force profile whereas the AbPB activity (in red) showed an inverse pattern. In other words, when the activation level of one muscle increased, the other decreased. As a consequence, for the right hand, the AbPB EMG activity was higher during SF0.5 than during SF1.5, whereas, the FDI EMG activity was higher during SF1.5 than during SF0.5. This negative correlation of EMG activities was expected given the function of these muscles and is known as the trade-off synergy [52]. Concerning the left hand muscles, the activation levels of both muscles were stable, as expected given the stable force production of the precision grip of the left hand.

### Corticomuscular coherences

Figures 3B and C show the CMC results obtained for a typical subject in the bimanual condition. The time-frequency plots of the z-score CMC (TFP_CMC_) for FDI of the right hand (right side of Fig. 3B) showed identifiable beta-band CMC bursts during the static force periods SF1.5 and SF0.5, for both electrodes C3 (lying over left M1) and FCz (lying over the medial frontal region). For FDI of the left hand (left side of Fig. 3B)_,_ the TFP_CMC_ showed more or less a constant beta-band CMC along the whole trial for electrode C4 (lying over right M1) but no identifiable CMC bursts for electrode FCz. Similar TFP_CMC_ were found for AbPB of both hands (not shown).

The corresponding topographies, averaged over the frequency band with the maximum CMC values (i.e., 20–30 Hz for left hand FDI and 16–26 Hz for right hand) are shown in Figure 3C for SF1.5 and SF0.5. It can clearly be seen that for right hand FDI the highest z-score CMC values are found over the left primary motor cortex (*M1_L_*) and the medial frontal region (right side of Fig. 3C). For left hand FDI, the highest z-score CMC values were only found over the right primary motor cortex (M1_R_) (left side of Fig. 3C). One additional topography for left hand FDI is shown in Figure 3C (left side), averaged over the frequency band 20–30 Hz and between 2 and 4 s where the left hand force production was stable. One can notice that eventhough the z-score CMC value for M1_R_ for left hand FDI was similar to that found for M1_L_ for right hand FDI, the z-score CMC value over the medial frontal region is close to zero.

**Figure 4:**
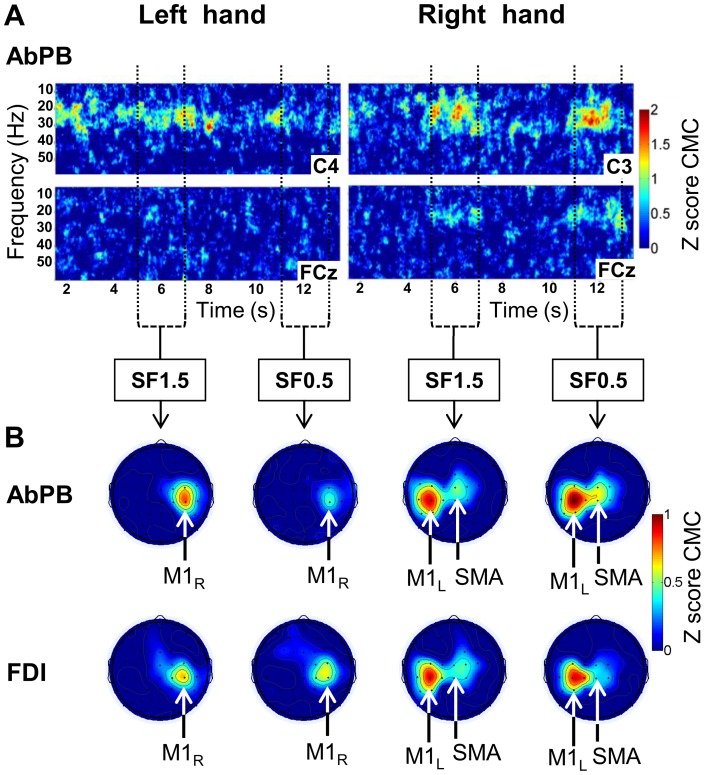
Corticomuscular coherence (CMC) obtained for left and right hand muscles in the bimanual condition. **A.** Grand average time-frequency plots of the z-score CMC between left hand AbPB *and* electrodes C4 and FCz (left side) and between right hand AbPB *and* electrodes C3 and FCz (right side). The color scale indicates the z-score CMC values, thresholded between 0 and 2 for clarity. The vertical axis represents the frequency scale ranging from 10 to 60 Hz. The horizontal axis represents the time scale ranging from 1.5 to 13.5 s (0 s, off-scale, corresponds to the beginning of the ascending ramp). The vertical dotted lines indicate the time windows over which we averaged to obtain the topographies. **B.** Grand average z-score CMC topographies for AbPB and FDI of left hand (left side) and right hand (right side), averaged over the frequency band 20–30 Hz and over 2 s of the periods of interest SF1.5 and SF0.5, respectively. The color scale indicates the z-score CMC values, thresholded between 0 and 1 for clarity. *M1_R_*: right primary motor cortex. *M1_L_*: left primary motor cortex. *SMA*: supplementary motor area. The time-frequency plots for FDI were similar to those for AbPB (not shown).

Figure 4 summarizes the CMC results obtained for the bimanual condition. The grand average TFP_CMC_ for AbPB of the right hand (right side of Fig. 4A) showed identifiable beta-band CMC bursts during the static force periods SF1.5 and SF0.5, for both electrodes C3 (lying over M1_L_) and FCz (lying over the medial frontal region). For AbPB of the left hand (left side of Fig. 4A)_,_ the grand average TFP_CMC_ showed more or less a constant beta-band CMC along the whole trial for electrode C4 (lying over M1_R_) but no identifiable CMC bursts for electrode FCz. Similar grand average TFP_CMC_ were found for FDI of both hands (not shown).

The corresponding topographies, averaged over the frequency band with the maximum CMC values (i.e., 20–30 Hz) are shown in Figure 4B for SF1.5 and SF0.5 and each recorded muscle. It can clearly be seen that for both right hand FDI and AbPB the highest z-score CMC values are found over the left primary motor cortex (*M1_L_*) and the medial frontal region (right side of Fig. 4B), corresponding to the electrodes FC1, FC3, FC5, C1, C3, C5, CP1, CP3, CP5 for *M1_L_* and Fz, FCz, Cz for the medial frontal region. For left hand FDI and AbPB, the highest z-score CMC values were only found over the right primary motor cortex (M1_R_) (left side of Fig. 4B), corresponding to the electrodes FC2, FC4, FC6, C2, C4, C6, CP2, CP4, CP6.

No significant difference in the spatio-spectral domain was found between the two time-periods SF1.5 and SF0.5 for any of the muscles. Similarly, even though right hand AbPB and FDI had different relative activation levels during the two static force periods, for the two static force periods SF1.5 and SF0.5 no significant difference was found in the spatio-spectral domain between the two muscles. However, by comparing CMC values obtained for the right hand muscles with those obtained for the left hand muscles, for each muscle and for each static force production period, we found significant differences in beta range CMC values for electrodes overlying both *M1_L_* and the medial frontal region. The statistical results of the non-parametric permutation test are given in Table 1 for each muscle and each statistical force production period.

**Table 1:** Results of the non-parametric permutation test for comparison of CMC values between right and left hands.

		SF1.5		SF0.5	
		Frequency (Hz)	P value	Frequency (Hz)	P value
FDI	M1_L_	14–35	P<0.05	15–32	P<0.05
	SMA	15–28	P<0.05	17–25	P<0.05
AbPB	M1_L_	14–34	P<0.001	14–34	P<0.05
	SMA	16–25	P<0.001	15–29	P<0.05

**Figure 5:**
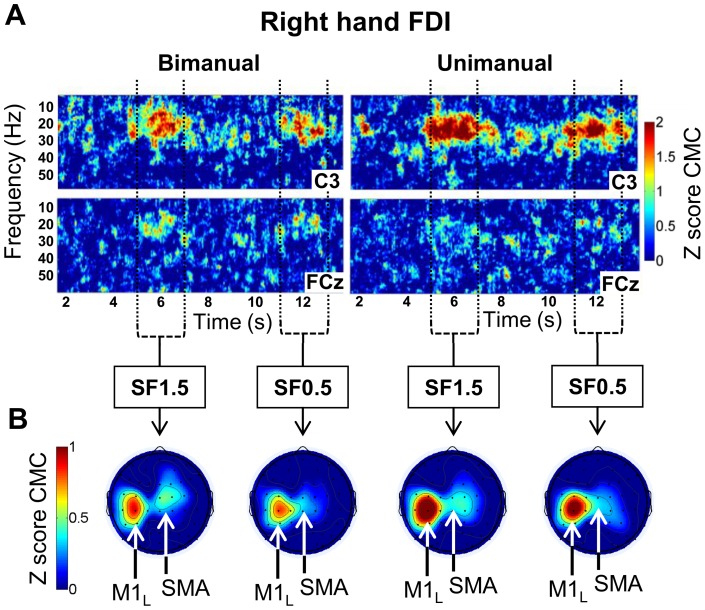
Corticomuscular coherence (CMC) obtained for right hand muscles for 5 subjects in the bimanual and unimanual conditions. **A.** Grand average time-frequency plots of the z-score CMC between right hand FDI and electrodes C3 and FCz in the bimanual condition (left side) and in the unimanual condition (right side). The color scale indicates the z-score CMC values, thresholded between 0 and 2 for clarity. The vertical axis represents the frequency scale ranging from 10 to 60 Hz. The horizontal axis represents the time scale ranging from 1.5 to 13.5 s (0 s, off-scale, corresponds to the beginning of the ascending ramp). The vertical dotted lines indicate the time windows over which we averaged to obtain the topographies. **B.** Grand average z-score CMC topographies averaged over the frequency band 20–30 Hz and over 2 s of the periods of interest SF1.5 and SF0.5 for right hand FDI obtained for the bimanual condition (left side) and the unimanual condition (right side). The color scale indicates the z-score CMC values, thresholded between 0 and 1 for clarity. *M1_R_*: right primary motor cortex. *M1_L_*: left primary motor cortex. *SMA*: supplementary motor area. Similar results were found for right AbPB (not shown).

Figure 5 shows the comparison of the CMC results between the unimanual and bimanual conditions, obtained for the 5 subjects who participated in both conditions. For both right hand muscles, statistical testing showed no significant difference in the spatio-spectral domain between the unimanual and bimanual conditions, neither for SF1.5 nor for SF0.5. Moreover, for the unimanual condition, as for the bimanual condition, neither the comparison between the two right hand muscles for each time-period, nor the comparison between the two time-periods SF1.5 and SF0.5 for each muscle, revealed a significant difference. In other words, the CMC values and their topographies were similar for the unimanual and bimanual conditions.

## Discussion

The aim of this experiment was to study the implication of the corticospinal (CS) projections originating from the supplementary motor area (SMA) in precise grip force control. The subjects were instructed to perform a visuomotor force tracking task with a precision grip, known to maximally mobilize CS projections [5]. We analyzed the corticomuscular coherence (CMC) between whole-scalp EEG signals and EMG signals of some intrinsic hand muscles. Since beta-band CMC reflects direct efferent neural transmission from cortical areas to muscles [31]–[33], studying beta-band CMC constitutes a non-invasive way to assess CS projections [25]–[27]. The bimanual condition was designed to impose a *cooperation between both hands to reach a common goal but with different roles for each hand* [43]. Indeed, the left hand had a postural role, holding the device without precision constraints in order to permit the right hand to precisely produce the required low forces. The unimanual control condition was performed to study whether the observed results for the right hand were related to the bimanual aspect of the task.

The behavioral results showed that both hands satisfactorily performed the task: the right hand force production varied in accordance with the target force profile while the left hand succeeded in holding the custom device. The between-trial variability of left hand grip force production, which was higher than that of right hand and, did not influence its performance, highlights bimanual cooperation, but not coordination, in this task. Moreover, the forces produced by both hands were in a similar force range, at least for the SF0.5 force period. The electrophysiological results showed clear beta-band CMC during the static force periods (SF1.5 and SF0.5). The CMC topographies showed that the beta-band CMC was spatially located for both hand muscles over their respective contralateral motor cortex (M1_R_ and M1_L_), which is in agreement with previously reported results obtained with EEG and MEG in human subjects [22], [42], [53]–[56] and with intra-cerebral recordings in monkeys [57]. It is now well-accepted that this beta-band CMC reflects the direct efferent neural transmission from primary motor cortex to contralateral distal hand muscles via the CS pathway. However, for the right hand muscles, and this is the main result of the present study, the beta-band CMC was also found to be located over the medial frontal region. This result strongly suggests that the CS projections from SMA are functional in the context of the present experimental protocol.

To our knowledge, functional coupling in the beta band frequency between the medial frontal region and hand muscles during a continuous isometric contraction has only been reported in a few studies, using either electrocorticogram [39] or EEG [37], [38], and was not found at the group level but only for some subjects. The dearth of reports in the literature concerning functional coupling between the medial frontal region and hand muscles is worth further discussion. The reason why our study was able to reveal this result is due to both methodological aspects and specificities of the present experimental protocol, which we will elaborate in the following. Then, we will end with some words concerning the type of CS projections from SMA that might be involved.

### Methodological aspects

Regarding the recording of EEG signals, the choice of the reference is known to influence the estimation of CMC [38]. In the present study, we used an average reference for which Mima and Hallett [38] also observed significant CMC on the medial frontal region in a task of weak tonic contraction of the right hand AbPB. Interestingly, applying current source density (CSD), a method used to achieve reference-free and spatially sharpened EEG, abolished this CMC over the medial frontal region. Therefore, one of the reasons why the CMC above the medial frontal area has been rarely reported in the literature is that the CSD was applied to EEG data in the majority of the studies in which subjects performed an isometric contraction task [31], [50], [55], [56], [58]. Another explanation could be the use of Cz as the reference electrode [27], [28] which diminishes the amplitude of the signal measured on the medial frontal area.

In order to explain the lack of CMC on the medial frontal region with the use of CSD, Mima and Hallett [38] proposed two possibilities. The first is that the significant CMC on the SMA region is the direct result of a volume conduction potential from the generator at the primary sensorimotor cortex which is correctly removed by the spatial filtering effect of CSD. The second is that it is due to a deep generator, such as SMA, that is blunted by the excessive spatial sharpening effect of CSD analysis. The results of our study are not in accordance with the first explanation. Indeed, the topographies in Figures 3 and 4 clearly show coherence between left hand AbPB and FDI *and* M1_R_ without CMC on the medial frontal region (see left side of Fig. 3C and 4B). This is particularly clear in the result for a typical subject (Fig. 3) for the 2–4 s period where for left hand FDI, we found CMC over M1_R_ (left side of Fig. 3C) with values comparable to those found over M1_L_ for right hand FDI during the periods of interest but without CMC over the medial frontal region. If the CMC on the medial frontal region was exclusively due to a volume conduction effect, significant CMC on this region would also be found for left hand muscles. Consequently, our results are in agreement with the second explanation and strongly suggest the existence of a deep generator leading to CMC on the medial frontal region.

The cingulate motor area (CMA) is also known to directly project on the spinal cord [10]–[12]. Since CMA is localized directly under SMA, the beta-band CMC found on the medial frontal region could also partly originate from CMA. Although this cannot be totally excluded, it seems highly improbable: spectral analysis was performed for each trial separately prior to averaging, and it is unlikely that the signal coming from such a deep structure (which, moreover, is localized underneath an active motor area) could be sufficiently detected by surface electrodes to give such significant CMC. Therefore, the present results strongly suggest that the significant beta-band CMC found on the medial frontal region mainly reflects neural communication between SMA and right hand intrinsic muscles.

### Specificities of the present protocol

Other reasons for the scarcity of reports on neural communication between SMA and intrinsic hand muscles may be related to the experimental protocols. If this communication only appears in some particular tasks, analysis of the protocols might reveal the functionality of the spinal projections from SMA. We will now highlight two specific aspects of the present protocol.

In the present bimanual condition, the finger grip configuration as well as the produced force levels were similar for both hands. However, we only found CMC between SMA and right hand muscles but not with left hand muscles. Hence, the difference must be due to aspects other than general precision grip control and static force production. Undeniably, the two hands performed a different behavioral task: The left hand had a postural role by “just” holding the device while the right hand performed precise force control based on continuous visual instruction and feedback on the force production. This difference was nicely reflected in the higher between-trial variability in force production of the left hand with respect to the right hand. Indeed, since neither instruction nor feedback was given concerning the force production of the left hand, the subjects paid little attention to the level of the force with which they gripped the device, leading to a higher variability in the force level between trials. This difference in the precision of the force control between both hands has likely led to the difference in CMC on SMA, suggesting that the communication between SMA and intrinsic hand muscles is related to precision of force control.

The relation between SMA activity and the precision of force control corroborates the results of a fMRI study of Kuhtz-Buschbeck and collaborators [18]. They found an increased activity in SMA when subjects gently held an object (i.e., with the lowest possible force level preventing the object from slipping) compared to a normal or firm hold. Since the gentle hold required a higher precision of force control, this strongly suggests that SMA activity increases with precision of the force control. Yet, fMRI can not reveal whether the increased activity in SMA is related to increased activity of neurons projecting to the spinal cord. Nevertheless, in a parallel fMRI and TMS study, we recently showed for M1 that both the BOLD response *and* the corticospinal control of the thumb-index grip increased with the level of precision of the force control [59]. If we suppose that this also holds in the study of Kuhtz-Buschbeck and collaborators [18], their results would be in line with our results, suggesting that communication between SMA and spinal motoneurons is related to the precision of force control.

Besides the precision of the force control, the way the target force was presented to the subjects might also have influenced the beta-band CMC over the SMA region. In a MEG study of Kilner and collaborators [26], subjects performed a task in some aspects similar to the one of the present study, i.e., matching right hand precision grip force production with that of a target force containing static force periods, ascending and descending ramps. But localization of the cortical sources with activity coherent with contralateral hand muscle activity only showed dipoles in M1, and not in SMA. Yet, an interesting difference between their protocol and the present one is the way the target force was presented to the subjects: the target force was presented by a box that moved up and down on the screen, and the visual feedback of the produced force was given by a square cursor which the subjects had to keep within the target box. Therefore, the subjects had no visual information concerning the upcoming variations of the target force, i.e. no information relating to the timing of force control. This type of visual presentation of the target force has been also used in other studies [e.g. 28]. In our study, the target force was a curve continuously visible in a ten-second window. As the horizontal position of the cursor was fixed in the middle of the screen, the subject was aware of the required force profile up to five seconds in advance. Interestingly, the implication of SMA in the planning of precision grip control has already been demonstrated in previous fMRI studies [17]. We propose that the CS communication between SMA and motoneurons of intrinsic hand muscles is related to the precision of force control, in particular when the target force profile modification can be anticipated.

One might argue that the hand dominance effect could have contributed to the present findings. If this had been the case, switching the role of the hands would have less engaged SMA. However, the present study included only right-handed participants, so producing a highly precise force is easier with the right hand than with the left hand: Witte and colleagues [60] and Kristeva and colleagues [27] showed the positive correlation between CMC and performance suggesting that since the present task would be more difficult with the left hand for right-handed subjects, it may lead to lower performance and therefore lower CMC values in the present study. As CS projections also exist from SMA to left hand muscles (e.g., [61]), it is highly probable that we may also find significant (after training) CMC between SMA and left hand when this latter one is performing high precision force control tasks. Moreover, a recent fMRI study [62] on right-handed subjects showed an implication of bilateral SMA in unimanual high-precision visuomotor force tracking tasks. Indeed, the authors found SMA to be active bilaterally, independently of the hand performing the task, which suggests an absence of hand dominance in the involvement of SMA.

The lack of difference in beta-band CMC between our unimanual and bimanual conditions are in line with the findings of the study mentioned above [62], showing equal implication of SMA in both unimanual and bimanual conditions. In the present study, whether the subjects held the device with their left hand or not, we found beta-band CMC between SMA and the right hand muscles. Although several previous fMRI studies have shown specific implication of SMA in bimanual tasks [34]–[36], the present results strongly suggest that the communication between SMA and right hand spinal motoneurons does not depend on the bimanual aspect of the task. Although the left hand had an indispensable role in the bimanual condition, the neural communication between SMA and the right hand is not affected by the left hand control. It is interesting to note that this finding again shows that the BOLD signal does not directly reflect the activity of CS neurons. Indeed, even if SMA is active bilaterally, we only found CMC with the right hand muscles, i.e. the hand performing the high precision force control task.

### Corticospinal versus cortico-motoneuronal projections from SMA

Corticomotoneuronal (CM) projections form a part of the overall corticospinal (CS) projection. Lemon and collaborators, in their review in 1998 [5], wrote that “although corticospinal outputs from M1 and SMA may act in parallel […], the direct cortico-motoneuronal influence from M1 upon hand and arm movements would appear to be much greater than that from SMA” (page 206). This expresses their doubts about the functionality of CM projections from SMA. It is true that in monkeys CM projections from SMA are less numerous and slower than those from M1 [11], [63]. Yet, since the CM pathway in primates has developed during evolution in a way correlated with the development of hand function, especially with the use of digits for prehensile purposes and for manipulation, differences in the organization of CS projections across species may well reflect differences in the functional contributions of the CS system [13]. Therefore, it might be possible that projections from SMA on hand muscle motoneurons are more developed in humans, with their higher ability to do fine manual manipulation. Interestingly, it has recently been shown that motor-evoked potentials (MEPs) can be evoked in contralateral distal hand muscles only 20 ms after transcranial magnetic stimulation (TMS) of human SMA [15], [16]. Since the latency of these MEPs was comparable to those obtained with TMS of M1, this result strongly suggests the presence of CM projections from SMA on spinal motoneurons of hand muscles. The neural communication between SMA and the hand muscles found in the present study might reflect the use of this CM pathway, but this remains to be confirmed.

## Conclusion

The present study showed significant beta-band coherences between the medial frontal scalp region and muscles of the right hand, involved in a precise force control task, but not with muscles of the left hand, involved in a postural task. We argued that this corticomuscular coherence (CMC) is generated by a deep source in SMA that could be revealed by the use of an average reference in the EEG acquisition. As beta-band CMC is well-known to reflect efferent neural transmission, this strongly suggests neural communication between SMA and motoneurons of intrinsic hand muscles. Since for the right hand no difference was found between the unimanual and bimanual conditions, this CS neural transmission from SMA seems to be unrelated to the bimanual aspect of the present task. Still, the bimanual condition, in which both hands produced similar forces with a similar grip configuration but with very different precision constraints, provided a means to demonstrate that the neural communication between SMA and the hand muscle motoneurons is related to the precision of force control. We conclude that the corticospinal projections from SMA become functional when manual force control requires high precision.
